# Publisher Correction: Detection of mosaic and population-level structural variants with Sniffles2

**DOI:** 10.1038/s41587-024-02141-2

**Published:** 2024-10

**Authors:** Moritz Smolka, Luis F. Paulin, Christopher M. Grochowski, Dominic W. Horner, Medhat Mahmoud, Sairam Behera, Ester Kalef-Ezra, Mira Gandhi, Karl Hong, Davut Pehlivan, Sonja W. Scholz, Claudia M. B. Carvalho, Christos Proukakis, Fritz J. Sedlazeck

In the version of the article initially published, Supplementary Tables 1–19 were missing. They are now included in the Supplementary Information.

